# A hybrid Stacking-SMOTE model for optimizing the prediction of autistic genes

**DOI:** 10.1186/s12859-023-05501-y

**Published:** 2023-10-06

**Authors:** Eman Ismail, Walaa Gad, Mohamed Hashem

**Affiliations:** Information Systems Department, Faculty of Computer and Information Sciences, Ain Shams University, Cairo, Egypt

**Keywords:** Gene similarity function, Gene prediction, Boosting techniques, Gene ontology, Ensemble learning, Stacking

## Abstract

**Purpose:**

Autism spectrum disorder(ASD) is a disease associated with the neurodevelopment of the brain. The autism spectrum can be observed in early childhood, where the symptoms of the disease usually appear in children within the first year of their life. Currently, ASD can only be diagnosed based on the apparent symptoms due to the lack of information on genes related to the disease. Therefore, in this paper, we need to predict the largest number of disease-causing genes for a better diagnosis.

**Methods:**

A hybrid stacking ensemble model with Synthetic Minority Oversampling TEchnique (Stack-SMOTE) is proposed to predict the genes associated with ASD. The proposed model uses the gene ontology database to measure the similarities between the genes using a hybrid gene similarity function(HGS). HGS is effective in measuring the similarity as it combines the features of information gain-based methods and graph-based methods. The proposed model solves the imbalanced ASD dataset problem using the Synthetic Minority Oversampling Technique (SMOTE), which generates synthetic data rather than duplicates the data to reduce the overfitting. Sequentially, a gradient boosting-based random forest classifier (GBBRF) is introduced as a new combination technique to enhance the prediction of ASD genes. Moreover, the GBBRF classifier combined with random forest(RF), k-nearest neighbor, support vector machine(SVM), and logistic regression(LR) to form the proposed Stacking-SMOTE model to optimize the prediction of ASD genes.

**Results:**

The proposed Stacking-SMOTE model is evaluated using the Simons Foundation Autism Research Initiative (SFARI) gene database and a set of candidates ASD genes.The results of the proposed model-based SMOTE outperform other reported undersampling and oversampling techniques. Sequentially, the results of GBBRF achieve higher accuracy than using the basic classifiers. Moreover, the experimental results show that the proposed Stacking-SMOTE model outperforms the existing ASD prediction models with approximately 95.5% accuracy.

**Conclusion:**

The proposed Stacking-SMOTE model demonstrates that SMOTE is effective in handling the autism imbalanced data. Sequentially, the integration between the gradient boosting and random forest classifier (GBBRF) support to build a robust stacking ensemble model(Stacking-SMOTE).

## Introduction

There are many diseases that particularly affect children, such as autism spectrum disorder (ASD) [1]. It may affect children at an early age, as it affects the child’s social behavior, and the child becomes isolated from the world and remains in his own world. The child with autism also suffers from difficulty in speaking, and his reactions and response to things become slow. Autism spectrum disorder is also an associated genetic defect that affects brain development. Therefore, It is important for early diagnosis and effective treatment of autistic people for a better life [2]. It is easy to identify people with autism through the apparent symptoms, but the genetic causes of the disease are very few. Therefore, we are interested to predict the genes associated with ASD for early diagnosis. Machine learning (ML) techniques are utilized by most reachers to predict the genes of the disease. Many studies have been done to identify the genes that cause autism, and hundreds of genetic causes have been discovered [3]. However, only 20% of the genes that cause autism have been discovered, and many genes remain undiscovered [4].

Most machine learning-based models suffer from an imbalanced distribution of the dataset, where most of the class samples belong to one class and a few samples belong to the other class. The most sample class is called the majority class, and the less one is called the minority class. Therefore, it is most important to learn from the imbalanced dataset, for better gene prediction as we need to predict the largest number of genes causing ASD. There are two ways to solve the imbalanced class distribution using undersampling techniques and oversampling techniques. Overall resampling techniques aim to learn the classifier not to bias to the majority class and reduce the model error. Undersampling techniques remove randomly selected instances from the majority class to make a balanced dataset. Oversampling techniques are two techniques, the first technique duplicated some samples from the minority class randomly, which may lead to an overfitting problem. The second technique is based on generating synthetic minority class samples such as s Synthetic Minority Oversampling TEchnique (SMOTE), Adaptive Synthetic Sampling Technique (ADASYN), SVM-SMOTE, and Borderline-SMOTE [5]. Moreover, there are more oversampling techniques that aim to create the most useful synthetic samples from the minority class giving weight to these samples representing their importance to the data such as MWMOTE [6], NI-MWMOTE [7], and IA-SUWO [8].

Supervised machine learning techniques are used to differentiate between disease genes and non-disease genes. Krishnan et al [9] built a weighted support vector machine (SVM) to predict the relationships between the brain genes and the genes associated with ASD. They also built a network to train their prediction model, which combined protein-to-protein interaction, gene expression, and gene regulatory network. They evaluated the model using Simons Foundation Autism Research Initiative(SFARI) database using the highest confidence genes. This model has limitations in using gene expression and protein-to-protein interactions as it can not represent the weak interactions that affect the model performance.

To overcome the limitations and disadvantages of the krishnan model, recent studies utilized gene ontology (GO) to calculate the similarity between the genes in their prediction model [10, 11]. GO is the largest source of information about the genes, which are categorized into three classes Molecular Function, Cellular Component, and Biological Process [12]. They are interested in genes that participate in only biological processes. In [11], different classification techniques are used to predict the genes associated with ASD, where the random forest classifier outperformed other classifiers using GO. Moreover, different semantic similarity functions are used to calculate the similarity between genes and construct the gene functional similarity matrix such as Resnik [13], Wang [14], and Relevance [15] methods.

Sequentially, in [16], machine learning techniques are used to predict the biomarkers of ASD genes. They studied the cell type of the brain associated with molecular pathology for ASD. Machine learning techniques are used to prioritize the highest confidence genes using the brain gene expression for a better predictive model to predict the cell type. ASD-Risk model was proposed in [17] to predict the risk genes of ASD. Support vector machine classifier is built using the gene expression profiles of ASD to predict ASD risk genes and define the brain temporospatial regions. Moreover, PANDA approach [18], built a network-based deep learning approach to predict ASD genes from the human genome. It used a gene-gene interaction network to build their model, which outperformed others classification techniques. In [19] different machine learning algorithms are used to effectively predict the early ASD traits in toddlers and adults.

Recently, some studies applied ensemble learning techniques [20–24] in their prediction models. PUStackNGly model is introduced in [20] to predict N-linked glycosylation based on stacking and bagging ensemble learning techniques. In [21] they applied different ensemble techniques such as stacking and voting to predict heart disease using different classifiers. ForecASD model is proposed in [22] to identify the risk genes of autism. They combined different networks in ensemble classifiers such as gene expression of the brain and other network data which affect the prediction of ASD risk genes. Moreover, HEC-ASD model [23] is proposed to predict ASD genes using gradient boosting ensemble learning techniques. They also proposed a new hybrid semantic similarity method(HGS) to measure the similarity between genes to construct the gene similarity matrix. Also, in [24], they build a machine learning-based model using different gene expression profiles of ASD data and network-based association genes to predict the novel ASD-associated genes. They utilized XGBoost classifier, NB, neural network, and RF to assess the performance of their model. XGBoost recorded the highest performance compared with other classifiers.

To overcome all the limitations of all proposed prediction models of ASD, we propose a hybrid Stacking-SMOTE model to predict the largest number of ASD genes using the following enhancements:

- Using a hybrid gene similarity function(HGS) that exceeds the other traditional similarity functions to measure the similarity between the genes effectively.
- Using SMOTE to handle the imbalanced dataset problem. It generates synthetic data samples from the original dataset without duplicating data which makes it effective to make a balanced dataset.
- Proposing a new classification technique that uses a gradient boosting technique based on random forest(GBBRF). It enhances the performance of the proposed model compared with the other traditional classifiers.
- Utilizing the stacking ensemble learning techniques to propose the Stacking-SMOTE model. It exploits the enhancement of the GBBRF combined with other classifiers to form a more robust prediction model.

The remaining of the paper is organized as follows: Section 2 describes the methods used to form the proposed Stacking-SMOTE model. The experimental results are presented in Section 3. Section 4 presents discussion and interpretation of the results. Sequentially, the conclusion is presented in section 5.

## Hybrid Stacking-SMOTE model

**Fig. 1:**
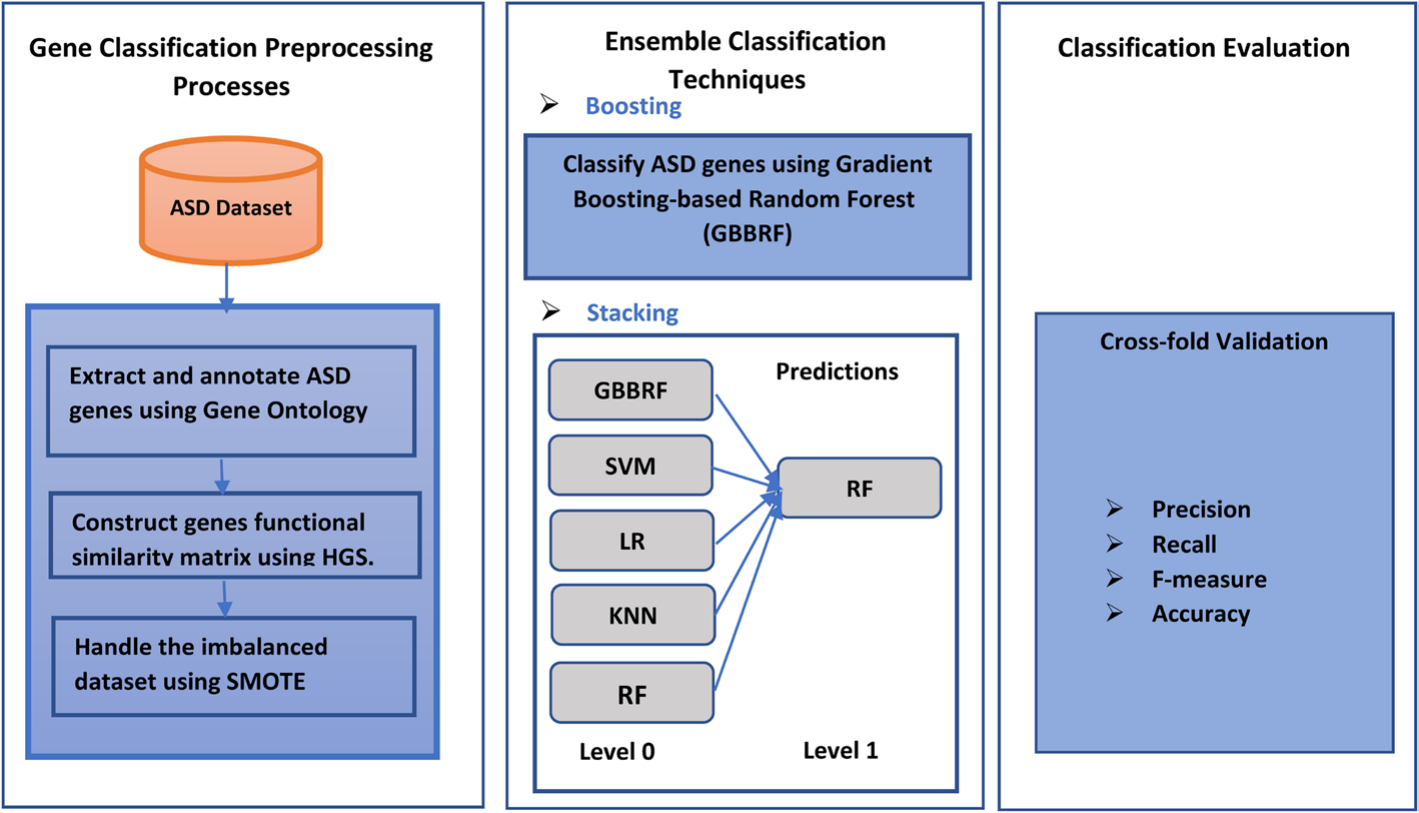
Proposed Stacking-SMOTE model framework to predict ASD genes

The proposed Stacking-SMOTE model framework is shown in detail in Fig.  1. It consists of three modules. The first module contains gene classification preprocessing processes, which concludes the extraction of all candidates ASD genes and generates the gene functional similarity matrix. Moreover, this module handles the problem of an imbalanced dataset associated with the SFARI database. The second module illustrates all required ensemble learning classification techniques used in the proposed model to optimize the prediction of ASD genes. The final module is the evaluation of the proposed model via cross-fold validation with different similarity measures.

### Gene classification preprocessing processes

Simons Foundation Autism Research Initiative (SFARI) database is used in this work https://gene.sfari.org/. It contains all candidate genes referring to autism spectrum disorder to predict new ASD genes. It is categorized into six classes. Categories 1,2,3, and 4 were only utilized in the analysis of the proposed model. There are three different preprocessing processes included in the proposed model. The first process extracts all these categories from the SFARI database and annotates all candidates ASD genes with gene ontology terms from Gene Ontology (GO) [25]. GO enriches the candidates ASD genes with semantic information to measure the similarity between genes. It is a hierarchical graph that represents all gene relations in the form of networks as nodes and edges. The nodes represent the gene terms and the edges represent the relations between terms. The term representation is classified into three different categories. The first category represents the molecular function (MF) of the happened activities, not interested in the causes of these activities and where they happen. The second category is the cellular component (CC), which represents the locations in the cell where the gene performs its function. The last category is the biological process(BP) which is the most important one responsible for the living organism, starting from its configuration and reaching the final product.

In this work, the proposed model utilizes only the biological process branch to annotate genes. The second process, the gene functional similarity matrix [26]is constructed based on a hybrid gene similarity function (HGS) [23]. There are different gene similarities functions such as Resnik, Relevance, and Wang. However, the proposed model is based on HGS, as it outperforms other methods [9, 10]. HGS measures the similarity between genes. It is a hybrid method between the Wang method and information gain(IG) based methods. It takes the benefits from IG of the term using only the number of its ancestors children rather than taking a long time searching in a large corpus file of IG of the term.

The gene functional similarity(GFS) matrix should be constructed first before classification using HGS. GFS is a matrix of rows and columns containing the candidate’s ASD genes and the cells between the genes filled with their semantic value of them. HGS method adapts GO to annotate the genes. At first, each gene was annotated with gene terms from GO. For example $g_1= {t_{11}, t_{12},.............., t_{1\,m}}$, $g_2= {t_{21}, t_{22},.............., t_{2n}}$, the semantic value of each term should be calculated against all terms of the other gene. Finally, the semantic similarity value between two genes is calculated by taking by mixing the semantic values between their terms using the average best-matching strategy[15]. After that, HGS measures the semantic similarity between two terms of GO using the following steps:

- Retrieve the directed a cycle graph (DAG) for each term, as an example, if we have a gene ontology term x its $DAG_X = (X, T_X, E_X)$, where $T_X$ is terms represents X and its ancestor nodes and $E_X$ is the edges that relate to these terms.
- Calculate the semantic value that represents the contribution of term X to its ancestors $S_X{(t)}$ using this formula is the same as Wang method if t = X then, $S_X{(t)} = 1$ else $S_X{(t)} = max (W* S_X{(t')} )$ where $t'$ is one of t childern
- Calculate the weight *W* for semantic value based on the number of term children and the type of its edges [23] using Eq. 1
  1 $$\begin{aligned} w = \dfrac{1}{number\;Of\;Childeren(t)+c} +d \end{aligned}$$

SFARI database is suffering from imbalanced distribution of its classes. It contains a large number of non ASD genes versus a small number of ASD genes. Therefore, the majority class is “Non-ASD”, which is much bigger than the minority class “ASD” making an imbalanced dataset. The problem with the imbalanced dataset that most machine learning techniques give biased results towards the majority class. Most of the time we are interested in the minority class as in this case we are interested in predicting the ASD genes. There are two techniques used to handle the imbalance problem, which are undersampling and oversampling. Undersampling is not the best solution as we remove some instances from the majority class to make a balance, but we are losing some data that may be important leading to decrease the model accuracy. Therefore, oversampling is chosen to best handle the imbalanced dataset problem. Synthetic Minority Over-sampling Technique (SMOTE) [27] is an oversampling technique used to handle this problem, which is represented as the third process as preprocessing techniques. Most oversampling techniques reuse some samples from the minority class, which may be duplicated some samples leading to an overfitting problem. Therefore, SMOTE technique creates new synthetic samples from the minority class to prevent overfitting. SMOTE is based on K-nearest neighbor(KNN) [28] algorithm. Algo.1 illustrates the SMOTE algorithm. The general idea of SMOTE algorithm is selecting a random sample from each sample neighbor and then creating a new synthetic sample from the minority class using linear interpolation between the two data samples. In this study the minority class is “ASD” and the majority class is “Non-ASD”. The detailed steps of the algorithm are explained as follows:
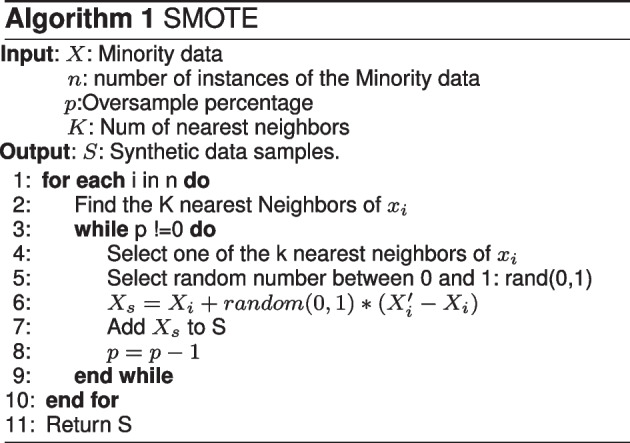

- Determine the vector of all minority class samples.
- For each sample $X_i$ in the vector, the Euclidean distance is used as the basic function to measure the distance between $X_i$ and all samples in the vector.
- Find the k-nearest neighbor samples of $X_i$, where k is an input to the SMOTE algorithm, k is equal to five.
- Resampling percentage is used as input to make a balance between the two classes, which depends on the number of data samples in the majority and the minority classes.
- Choose random samples $X'_i$ from the k-nearest neighbors of $X_i$.
- Generate new synthetic samples $X_s$ between $X_i$. and each sample $X'_i$ using the equation in algo.1, where random(0,1) is a random value between 0 and 1.
- Repeat the following steps until the data become balanced.

### Ensemble classification techniques

Ensemble learning techniques are based on machine learning methods that combine different predictive models to form a robust model. These techniques decrease the bias, and the variance and increase the performance of the predictive model. Ensemble classification techniques are used to build the proposed model using two different techniques: boosting and stacking techniques.

#### Boosting techique

Boosting is an ensemble learning technique. Boosting structure is built sequentially as it combines a set of weak learners for a more robust one. Each weak learner learns from the error of the previous weak learner. Gradient boosting is utilized in the proposed model to predict ASD genes. It is the most robust machine learning algorithm as it minimizes the total error of the proposed model. It worked iteratively, in each iteration it tries to minimize the loss function of the proposed model. The gradient boosting algorithm [29] is built based on three main components. The first component is the loss function, which must have a derivative. The loss function represents the efficiency of the predicted model, which is the difference between the actual value and the predicted one. The second important component is the weak learner, it is used to train the model but with low accuracy with high error. The weak learner is a simple decision tree called decision stumps. The third component is the additive model, which means in each iteration for adding a tree, the proposed gradient boosting model seeks to reduce the error forming a more robust model.

In this work, a gradient boosting-based random forest classifier(GBBRF) is proposed to improve the prediction accuracy. Random forest classifier is used to train the gradient boosting model as a weak learner either than decision stumps. This combination gained high performed model with less error.

#### A. random forest(RF)

RF is a meta-classifier, which consider one of the ensemble classification techniques. It is the most powerful classifier used in different machine learning classification techniques worked for high dimensional data. It builds upon ensemble decision trees using different subsampling on data creating different decisions. It is a bagging technique that is based on the bootstrap aggregation method. Bootstrapping method takes samples from the dataset with replacement to build decision tree. Each decision tree is split using entropy and information gain methods. These bootstrapping samples are combined and aggregated to form a random forest tree which is called the bagging method.

#### B. Gradient boosting based on random forest(GBBRF)

GBBRF is proposed as a new combination technique between gradient boosting and random forest. Random forest is used to be the base weak learner for the gradient boosting method. This combination helps to decrease the variance and the bias, forming a predictive model with less error. The algorithm steps of the proposed GBBRF technique in Algo.2. GBBRF method utilizes the log loss function to minimize the total error of the proposed model as in Eq. 2

2 $$\begin{aligned} L_{log\;loss} = -\frac{1}{N}\sum \limits _{i=1}^{N}y_{i}*log(p(y_{i}))+(1-y_{i})*log(1-p(y_{i})) \end{aligned}$$

In the first step, we initialize the model with constant value $F_{0}(X)$ as in Algo.2. L is the loss function [30] where $y_i$ is the predicted value and gamma is the log(odds) value. After that, a pseudo residual value is calculated for each constructed tree which is the difference between the observed value and the predicted one. Moreover, terminal nodes are created for each tree. Afterthat, the gamma values are calculated for each leaf in the tree and the summation should be the values that minimize the loss function. In the proposed GBBRF method, some regularization parameters are used to decrease the overfitting, such as the max depth of the tree, and the learning rate is used to adjust the model moving to the best results concerning the loss function.
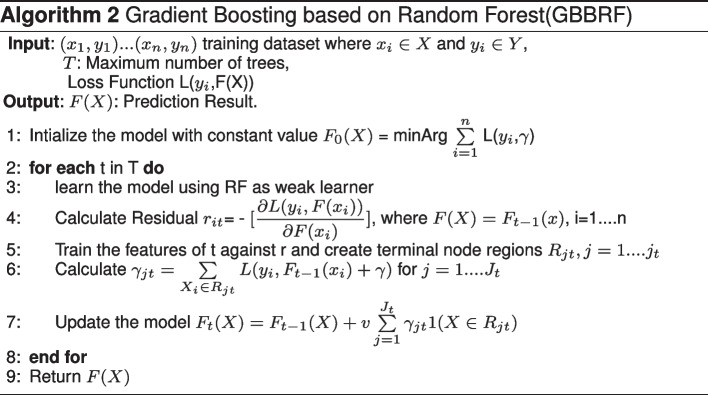


#### Stacking techique

Stacking ensemble learning builds a more robust prediction model utilizing the prediction of well performed prediction models. Therefore, a stacked-based prediction model is expected to outperform the individal classification model [11]. The proposed Stacking-SMOTE model uses the proposed GBBRF, Support Vector Machine (SVM), Logistic Regression(LR), K-nearest neighbor(KNN), and Random Forest(RF) to be the bases classifiers of the learner models.

**Fig. 2:**
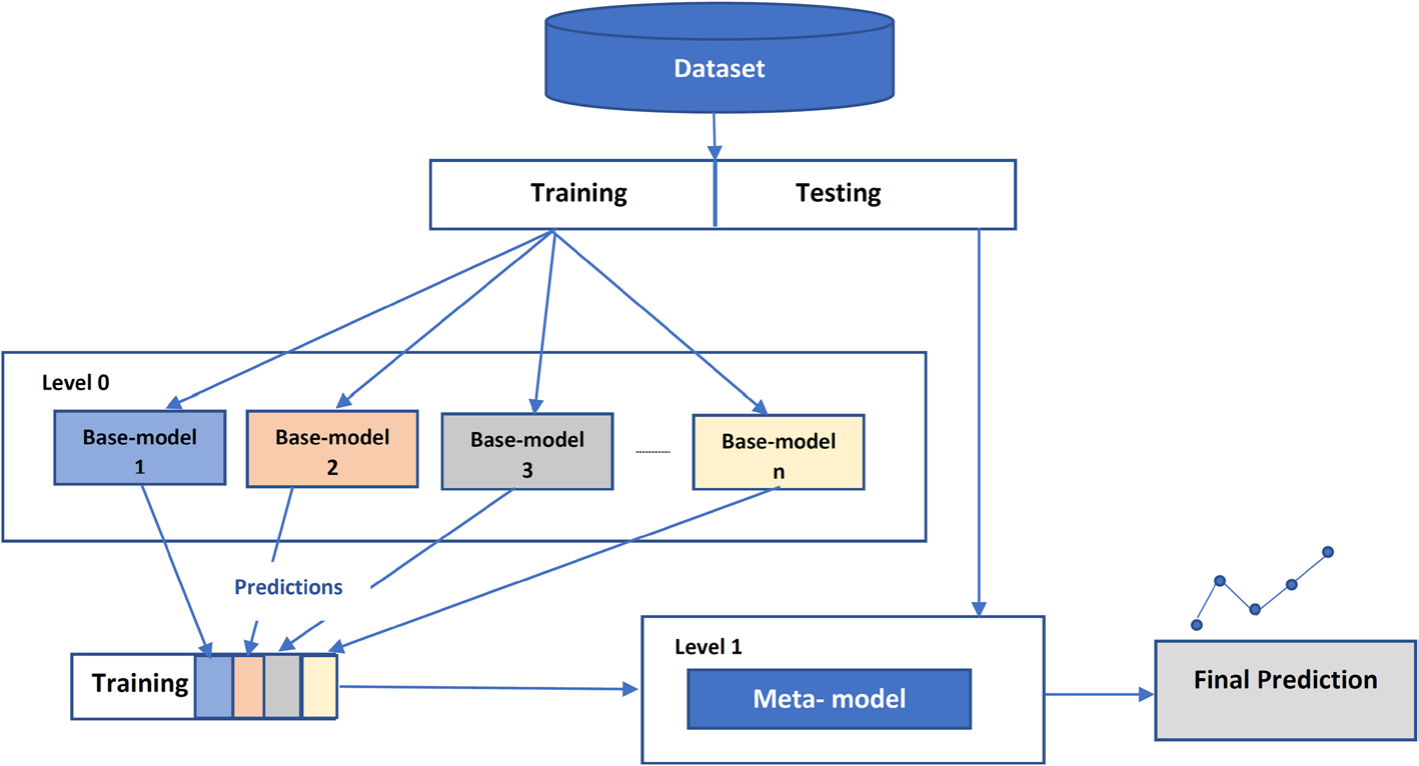
Stacking model Schema to predict ASD genes

Stacking-based model architecture is shown in Fig.  2 which concludes the following steps:

- Use K cross-fold validation to split the dataset to train and test sets.
- Use the k-1 sets to fit the base models and the last k for prediction.
- For each training set, a set of base learner models are trained in this set and evaluated on the test set.
- The outputs of the predictions from the base learners models are used as input for the final prediction model used in level 1.
- Level 1 model makes prediction on the test set as the final output of the Stacking-SMOTE prediction model.

Different classifiers are used as the base learner models such as SVM, KNN, RF, LR, and the proposed GBBRF classifier. SVM is used in most bioinformatics classification models. It aims to find the most suitable hyperplane to correctly classify the data. LR is suitable in binary classification problems as in our case “ASD” and “Non-ASD”. RF and KNN are also suitable in binary classification and perform well in most bioinformatics problems.

## Experimental results

### Database description

Simons Foundation Autism Research Initiative(SFARI) is used to assess the performance of the proposed model. SFARI is a gene database that contains up-to-date genes associated with autism spectrum disorder (ASD) results from research related to autism disease. It gives a score to every gene that represents the strength of the association between these genes and autism. The gene score is categorized into different categories, categories one and two represent the highest confidence genes(HCG) related to ASD, and categories three and four are the lowest confidence genes(LCG). These categories are included in the analysis of the proposed model. Moreover, any syndrome gene [31]that gets a score for these categories is also included in the analysis. SFARI database contains a total of 990 genes related to ASD, the highest confidence genes(HCG) counted 82 genes from them, 506 genes are considered to be the lowest confidence genes(LCG), but the rest genes from 990 are excluded from the analysis. These excluded genes may be genes with no score or have a score not included in categories 1,2,3,4. Moreover, 1189 non-mental genes [9] from (OMIM) are utilized in the analysis of the proposed Stacking-SMOTE model which is considered in the negative class “Non-ASD”.

### Stacking-SMOTE model evaluation

K Cross-fold validation technique [32] is used to evaluate the proposed model using SFARI database and a set of non-mental genes from krishnan[9]. It divides the dataset into k subsets. It uses k-1 subsets for training and the last subset for testing, where all subsets are equal in size. Stratified five cross-fold validation is used for model assessment. Therefore, four folds are used as training and the last fold for testing, then repeat this process five times and exclude different subsets each time for testing. Moreover, five different evaluation metrics [33] are used to evaluate the performance of the proposed model, which are precision, recall, f-measure, accuracy, and area under the curve- Receiver Operator Characteristic(AUC-ROC)[34]. All these metrics depend on the result confusion matrix, which is a table that combines four different values, the actual and the predicted values. The first is true positive(TP) means that the predicted value is positive and it is actually positive. The second is true negative (TN) means that the predicted value is negative and it is actually negative. The third is false positive (FP) means that the predicted value is positive and it is actually negative. The fourth is a false negative(FN) means that the predicted value is negative and it is actually positive.

Model accuracy is used to measure the number of corrected predictions using Eq. 3, which is the number of corrected predictions divided by the total number of predictions. Precision is the number of corrected positive predictions divided by the total number of positive predictions as in Eq. 4. Recall is the number of corrected positive predictions divided by the total number of relevant predictions as in Eq. 5. F-measure is the combination between recall and precision metric using Eq. 6. Moreover, AUC-ROC is the probability of the area under the curve that is drawn between the rate of true positive and the rate of false positive in different thresholds. The higher AUC means that the model is effective to distinguish between the classes of the dataset.

3 $$\begin{aligned} Accuracy= & {} \frac{TP + TN}{TP + TN + FP + FN} \end{aligned}$$

4 $$\begin{aligned} Precision= & {} \frac{TP}{TP + FP} \end{aligned}$$

5 $$\begin{aligned} Recall= & {} \frac{TP}{TP + FN} \end{aligned}$$

6 $$\begin{aligned} F-measure= & {} 2*\frac{Recall*Precision}{Recall + Precision} \end{aligned}$$

### Results

#### Results of the proposed model using SMOTE and other reported resampling techniques

Different classifiers are used to assess the performance of the proposed model such as NB, RF, SVM, and KNN. The results include a comparison between the performance of the model using SMOTE and other resampling techniques “RUS” and “SMOTE-RUS” reported in [23] to handle the imbalanced dataset. The comparison shows the results in terms of accuracy, precision, recall, and f-measure in Table 1. Two methods are used to build the gene functional similarity matrix. The first one uses the highest confidence genes and all non-mental genes proposed by Krishnan et al [9], and the second one uses the highest and lowest confidence genes with all non-mental genes. The results in [23] showed that the highest confidence genes with non-mental outperform the other method. Therefore, the proposed model utilizes the first method to enhance and optimize the process of autism gene prediction. Moreover, the hybrid gene similarity function (HGS)[23] is used as the base similarity function to measure the similarity between the genes in the proposed model. HGS outperforms the others gene similarity functions such as Resnik, Wang, and Relevance.

**Table 1:** Comparison between the proposed model-based SMOTE and RUS-based model using different classifiers

Classifiers	Evaluation metrics	RUS%	SMOTE-RUS%	SMOTE %
NB	Accuracy	74.8	68.6	76.1
	Precision	77.9	71.8	83.6
	Recall	74.9	68.6	76.0
	F-measure	76.2	69.8	79.0
RF	Accuracy	84.2	86.3	90.7
	Precision	79.7	86.4	90.0
	Recall	84.2	86.3	90.7
	F-measure	79.5	85.3	88.8
SVM	Accuracy	71.5	81.9	88.4
	Precision	71.2	82.7	90.2
	Recall	71.5	82.0	88.4
	F-measure	71.3	82.3	89.1
KNN	Accuracy	79.4	87.9	92.2
	Precision	79.8	90.1	94.1
	Recall	79.4	87.9	92.5
	F-measure	79.6	88.4	93.0

SMOTE creates synthetic samples using the five nearest neighbors for all the minority samples to handle the imbalanced SFARI database. After that. we randomize the order of the instances to get reasonable results when applying the k cross-fold validation technique. The results of the proposed model based on SMOTE outperform the model using random undersampling technique(RUS) [23]. Undersampling technique “RUS” is just a straightforward method as it deletes some examples from the majority class (Non-ASD). This technique is less effective when we deal with large data as it loses large valuable data when deleting some samples. SMOTE-RUS technique is a hybrid resampling method, Which handles the problem of imbalanced dataset using SMOTE to oversamling the minority class and apply random undersampling (RUS) in the majority class as in [35]. The results of RUS technique have the lowest accuracy compared to other resample techniques, which indicates that random undersampling technique is not the best choice in this case. RUS deletes randomly samples from the majority class, which may have important information. Sequentially, we applied SMOTE-RUS technique [35] that combines the benfits form SMOTE and decrease the effect of RUS in our proposed model, but it enhances the results a little bit. SMOTE is the best choice in our proposed model. It reaches an improved accuracy of 90% using the Random Forest(RF) classifier compared to the highest value of RF using random undersampling in the HEC model which is 84%. RF classifier and KNN showed the highest classifier performance rather than other classifiers.

#### Results of the proposed boosting technique (GBBRF)

**Fig. 3:**
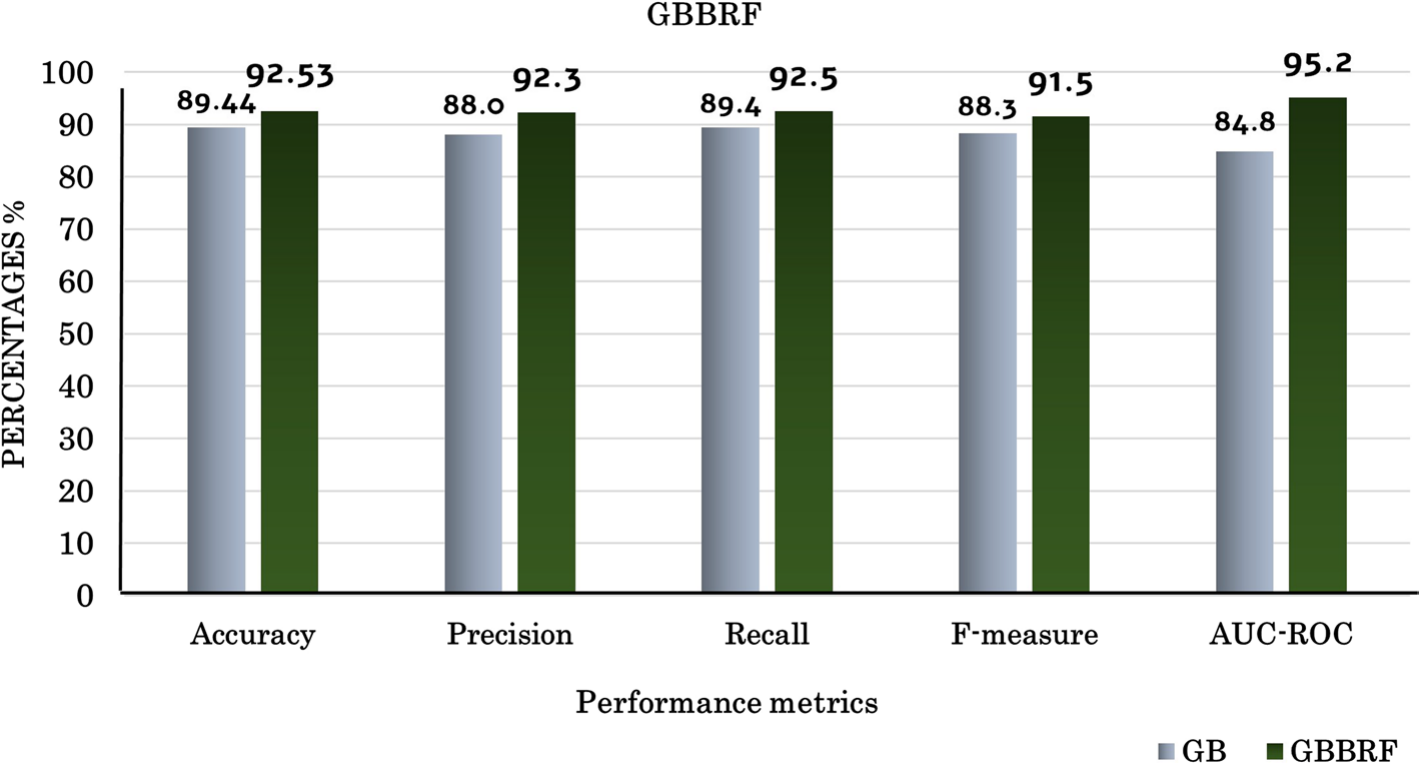
Comparison between the proposed GBBRF and GB ensemble techniques

Ensemble learning techniques are exploited to improve the performance of the proposed model. The proposed gradient boosting-based random forest(GBBRF) technique uses the hybrid gene functional similarity(HGS) function [23] to measure the similarity between genes and uses SMOTE to make a balanced dataset. GBBRF utilizes the random forest classifier to apply gradient boosting rather than the trivial gradient boosting (GB) technique which is based on decision stumps. Figure 3 shows the comparison between the proposed technique GBBRF and trivial GB in terms of Accuracy, Precision, Recall, F-measure, and AUC-ROC. The proposed GBBRF uses regularization parameters as learning rate equal 0.1, lambda equal 5, depth of individual tree equal 3, and num of trees = 500. The results from the combination between the gradient boosting and random forest classifier GBBRF show an increase in the model accuracy rather than GB. The accuracy reaches 92.5% using GBBRF compared to 89.44 % using GB. Using the random forest classifier to build the gradient boosting and using the regularization parameters prevent the model overfitting and increase the performance of the model. Random forest with gradient boosting achieves higher performance than trivial gradient boosting using deep trees. RF creates different decision trees independent of each other and randomizes the construction of the decision trees to have a variety of predictions. Therefore,the combination of RF and gradient boosting is better than using a simple decision tree, as it minimizes the model error and prevents overfitting.

#### Results of the proposed model-based stacking technique (Stacking-SMOTE)

Different classifiers combinations are used to form a robust Stacking-SMOTE model. These classifiers are RF[36], SVM[37], NB[38], KNN[28], LR[39], and the proposed GBBRF. These classifiers are tested and evaluated on the reported dateset using stratified five cross-fold validation. The results of these classifiers 1 show that GBBRF, KNN, and RF have the highest performance among the others classifiers. Therefore, GBBRF, KNN, and RF classifiers are chosen to be in the combination for level 1 and all used classifiers are made the combination for level 0 as shown in Table 2.

**Table 2:** Different stacking combination

Stacking number	Level 1	Level 0
Stacking 1	RF	NB, SVM, KNN, RF.
Stacking 2	GBBRF	RF, KNN, SVM, LR, GBBRF.
Stacking 3	RF	GBBRF, NB, SVM, KNN, RF
Stacking 4	RF	GBBRf, LR, SVM, KNN, RF
Stacking 5	KNN	GBBRF, LR, SVM, KNN, RF

In the proposed stacking model, the output of all sub-models in level 0 is used as an input to learn the model used in level 1. Level 1 model combines the input predictions and form a robust prediction model. Sequentially, the five prediction stacking models in Table 2 are evaluated on the SFARI dataset and the set of non-mental genes. The results in Table 3 shows that all stacking combinations show higher performance than using a single predictive classifier. Stacking 5 gets the lowest accuracy around 92.6% using KNN as level 1 model compared with other stacking that used RF. This indicates that KNN is not the best choice and RF works better in predicting the output prediction of the model. Stacking 3 and 4 outperform other stacking models and stacking 3 shows also the highest AUC-ROC. The higher AUC-ROC indicates the highest model performance. Therefore, stacking 3 is the best combination used to build the proposed Stacking-SMOTE model. Table 3 represents the performance of the stacking model combinations in terms of precision, recall, f-measure, AUC-ROC, and accuracy. The proposed Stacking-SMOTE model reaches an accuracy around 95.5%.

**Table 3:** The results of different stacking combinations

Stacking number	Precision	Recall	F-measure	AUC-ROC	Accuracy
Stacking 1	94.90	95.00	94.90	94.90	94.99
Stacking 2	94.60	94.80	94.70	95.20	94.76
Stacking 3	95.20	95.40	95.30	94.80	95.37
Stacking 4	95.20	95.40	95.30	94.70	95.37
Stacking 5	93.80	92.60	92.70	84.10	92.60

#### Comparison with other ASD gene prediction models

**Fig. 4:**
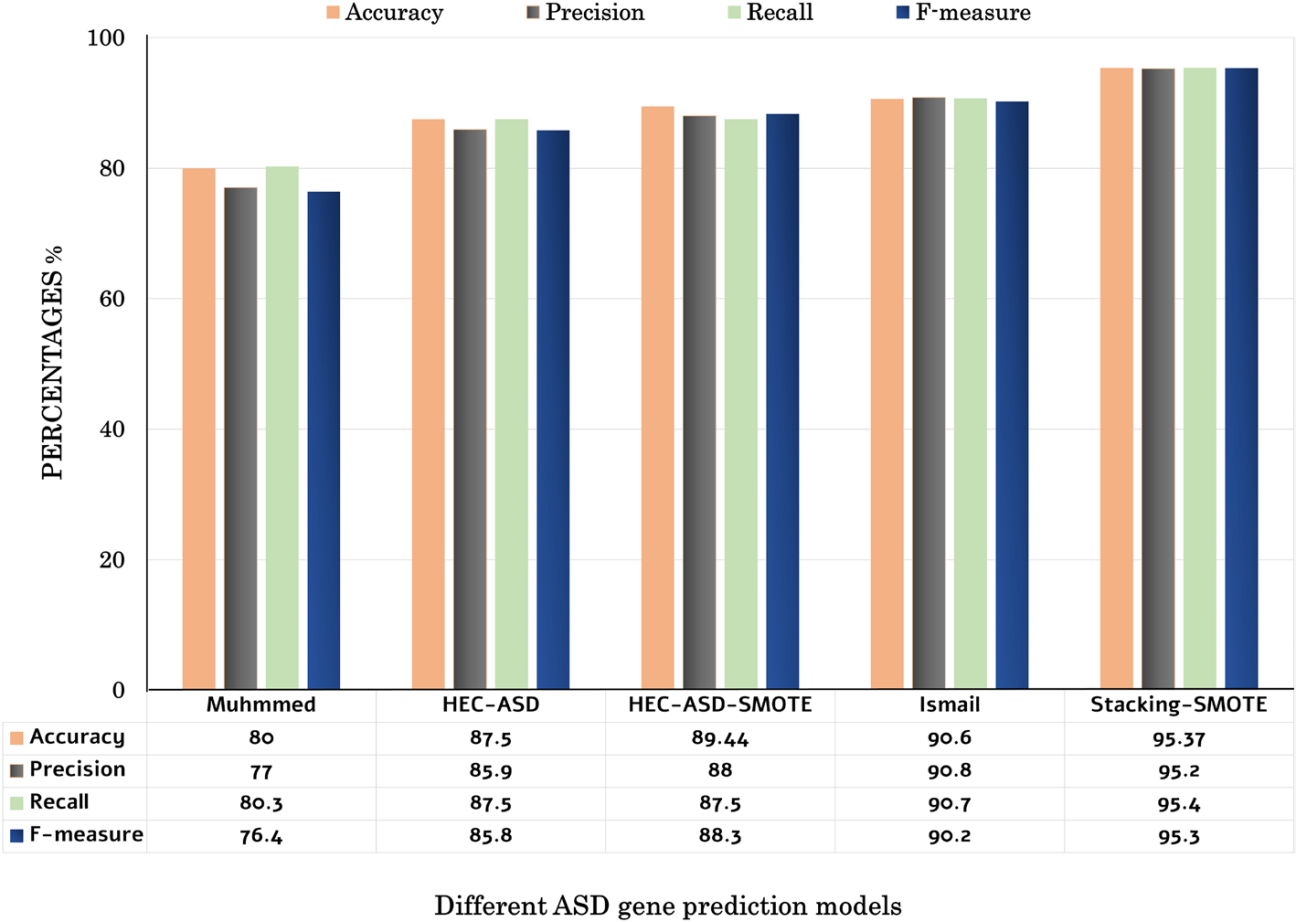
Comparison between the proposed Stacking-SMOTE model and the other mentioned prediction models

**Fig. 5:**
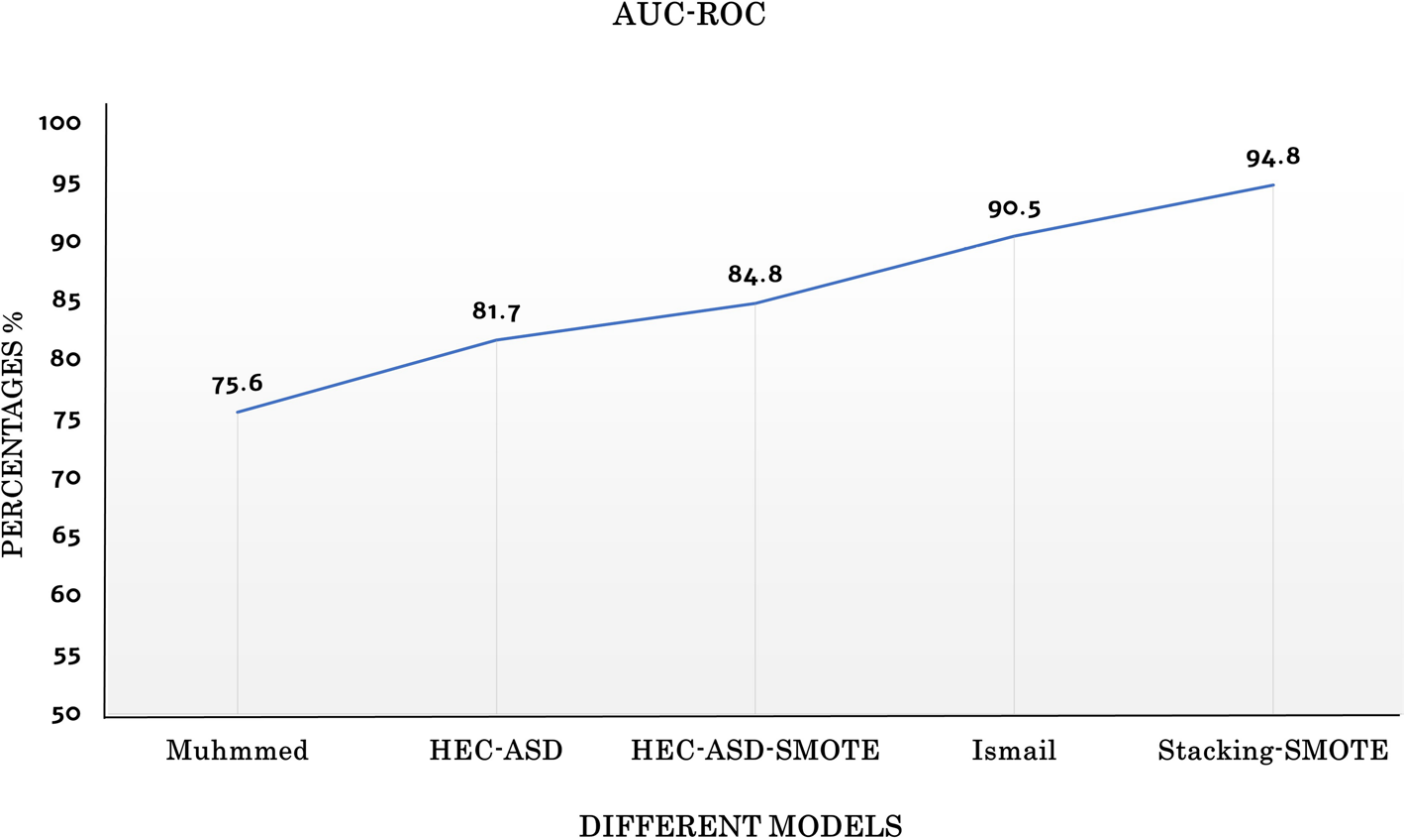
Comparison between the proposed Stacking-SMOTE model and other mentioned prediction models interms of AUC-ROC

The performance of the proposed Stacking-SMOTE model is evaluated with other models in [10, 23]. These mentioned prediction models are utilized in the comparison as they have the same target to predict ASD genes. They also use the same SFARI database for evaluating their models. Figure 4 shows a detailed comparison between the proposed Stacking-SMOTE model and the other prediction models. The comparison results are in terms of accuracy, precision, recall, and f-measure. In [10] they use gene ontology and use basic classifiers to predict the ASD gene using different semantic similarity functions to measure the similarity between the genes. HEC-ASD model that was proposed in [23] utilizes gene ontology to annotate the candidates ASD genes and proposed a new hybrid method (HGS) to measure the similarity between the genes. But this model uses the trivial solution to solve the imbalanced SFARI dataset problem. HEC-ASD is an ensemble-based model that used gradient boosting techniques to predict ASD genes. This HEC-ASD model gained high performance than krishnan et al. In our work, SMOTE is used to solve the imbalanced dataset problem, which enhances the performance of the prediction model. Therefore, we apply SMOTE to HEC-ASD model[23], and the results in Fig.  4 show that the HEC-ASD based on SMOTE “HEC-ASD-SMOTE” outperform the results of the HEC-ASD model. Moreover, the proposed Stacking-SMOTE model outperforms all other mentioned models, which reaches an accuracy of 95.5%. Figure 5 shows the comparison between the proposed Stacking-SMOTE model and other models in terms of AUC-ROC. The results of AUC-ROC show high improvement than the other models, which reflects the ability of the proposed Stacking-SMOTE model in distinguishing between the ASD genes and Non-ASD genes.

Moreover, we compare the performance of the proposed Stack-SMOTE model with other reported models in [9, 18, 24] as in Table 4. They used different data sources to predict the genes associated with ASD. The proposed Stacking-SMOTE model reaches an accuracy around 95.5% compared to Krishnan et al [9] which reached an accuracy around 73 %. In [9] they use gene regulatory networks and protein-to-protein networks to predict genes related to ASD. They used machine learning techniques using a weighted support vector machine to predict ASD-related genes. They use also cross-fold validation to evaluate their model. In [24], they build a machine learning-based model using different gene expression profiles of ASD gata and network-based association genes to predict the novel ASD association genes. They utilized XGBoost classifier that reaches the highest AUC around 82.4% to assess their model. Also, we compare the proposed model with a deep learning model (PANDA)to predict the ASD genes. They build their model using the human molecular interaction network to train a deep learning model using SFARI database and Online Mendelian Inheritance in Man (OMIM). Our proposed model accuracy outperforms PANDA model which rearches an accuracy around 89%. This comparison refers to the importance of using GO as a knowledge base of gene to better measuring the gene similarities as we utilized it in our proposed model.

**Table 4:** Comparison between different models using different data sources

Prediction model	Performance measure (AUC)	Classifier
Krishnan [9]	0.73	Weighted SVM
Suratanee [24]	0.824	XGBoost
Yu Zhang [18]	0.89	PANDA(Deeplearning)
Stacking-SMOTE	0.948	Stacking ensemble

## Discussion and interpretation

Gene prediction is one of the most important topics we must care about it, especially in the case of autism disease. As there are few genes detected in autism. Moreover, in most bioinformatics fields as in autism, the imbalanced dataset is one of the issues that should take care. Therefore, we proposed machine learning-based model to predict the genes causing ASD. We trained different classifiers such as RF, SVM, KNN, and NB using the generated matrix of the highest confidence genes HCG+non-mental genes. In this paper, SMOTE is utilized to solve the imbalanced dataset problem, which is more effective than other resampling techniques used [10, 23]. RF and KNN classifiers got the highest accuracy than other classifiers. The results of the proposed model using SMOTE are compared with other reported resampling techniques (SMOTE-RUS and RUS). The results of SMOTE outperform the other methods, which reach an accuracy of 90.7% using RF classifier. Moreover, this paper proposes a hybrid Stacking-SMOTE model to optimize the prediction of ASD genes. This model achieves high improvement in predicting the genes related to autism spectrum disorder (ASD) disease. The main improvement is summarized in the following point:

- Use SMOTE techniques to handle the imbalanced SFARI dataset. It is an intelligence technique, which generates synthetic data samples relative to the original minority samples rather than duplicates data samples as in the other resampling techniques.
- Utilizes the proposed hybrid gene similarity function (HGS)[23] to measure the semantic similarity between genes. It improves the prediction model rather than using Wang, Relevance, and Resnik similarity methods.
- Proposing ensemble learning technique using gradient boosting technique based on random forest(GBBRF). The combination of these techniques increases the prediction model performance more than using single classifiers.
- Proposing a hybrid Stacking-SMOTE model that combines the advantages of SMOTE in Stacking model. It uses different classification techniques combined with GBBRF technique.

The proposed GBBRF method enhances the performance of the proposed model. We compare the results of GBBRF against trivial gradient boosting, it increase the performance with 3%. It reaches an accuracy 92.5% compared to 89.4%. Moreover, it increases the AUC-ROC more that reflects the efficiency of the proposed model in differentiating between ASD and Non-ASD genes.

The proposed Stacking-SMOTE model optimizes the performance of the ASD genes prediction model rather than the reported models in [10, 23]. The proposed model results outperform other prediction models as we reach an accuracy of 95.5%. This reflects the importance of using ensemble stacking techniques rather than using a single prediction model. Moreover, the proposed Stacking-SMOTE model is compared with other models that uses different datasources such as gene expression profiles and gene regulatory network [9, 24]

In this article, We prove that GO is efficiency in measuring the gene functional similarities than using other methods. Therefore, the performance of the state-of-art methods to identify The ASD genes can be improved using GO annotations. The limitation of the proposed model is that a small number of genes do not have annotations in GO. Therefore, it expected in the future work, integrating the GO with another type of datasources such as gene expression or protein-to-protein interaction network will improve the performance of the prediction model.

## Conclusion

Autism spectrum disorders (ASDs) have become more prevalent among children lately, which is a complex genetic disease. Therefore, in this paper, we propose a hybrid model based on Synthetic Minority Oversampling TEchnique (SMOTE) and stacking ensemble learning technique. A set of candidates ASD genes is extracted from SFARI database and preprocessed using SMOTE to handle the imbalanced dataset. The stacking ensemble technique combines support vector machine(SVM), k-nearest neighbor(KNN), logistic regression(LR), random forest(RF) classifiers and the proposed gradient boosting-based random forest(GBBRF) to form a more robust prediction model. GBBRF is effective in optimizing the performance of the proposed hybrid model, which outperforms the other basic classifiers. Moreover, using SMOTE to handle the imbalanced dataset and utilizing our proposed HGS similarity function in measuring the similarity between the genes result in increasing the performance of the proposed model compared to others models. The results of the proposed hybrid Stacking-SMOTE model get the highest performance of 95.5% accuracy, which outperforms other techniques. This indicates the importance of using stacking ensemble technique to form a more robust prediction model than using individual classification models.

## Data Availability

The Simons Foundation Autism Research Initiative (SFARI) gene database, which analyzed during the current study, is available at https://gene.sfari.org/.
